# Aesculin based glucosamine-6-phosphate synthase inhibitors as novel preservatives for food and pharmaceutical products: in-silico studies, antioxidant, antimicrobial and preservative efficacy evaluation

**DOI:** 10.1186/s13065-021-00769-8

**Published:** 2021-07-27

**Authors:** Amit Lather, Sunil Sharma, Anurag Khatkar

**Affiliations:** 1 Laboratory for Preservation Technology, and Enzyme Inhibition Studies, Faculty of Pharmaceutical Sciences, Maharshi Dayanand University, Rohtak, Haryana India; 2 Department of Pharmaceutical Sciences, G.J.U.S.&T., Hisar, India

**Keywords:** Aesculin, Parabens, G-6-P synthase, Docking, Preservative

## Abstract

**Background:**

Presently available chemical based synthetic preservative have emerged with various side effects, so the aspiration of natural and side effect free novel preservative has been greatly increased. As the natural preservative exhibit poor side effect with improved preservative efficacy. The recent development in computational studies leads advancement in drug designing and discovery of novel glucosamine-6-phosphate synthase (G-6-P synthase) inhibition based natural antimicrobial preservatives. Here, selected aesculin derivatives were screened for G-6-P synthase inhibition via docking study and evaluated for antioxidant, antimicrobial, preservative efficacy as well stability study.

**Results:**

Modified aesculin derivatives were designed, synthesized and showed potent G-6-P synthase inhibition with remarkable antimicrobial, antioxidant, preservative efficacy and stability study. The molecular docking with target pdb id 1moq from G-6-P synthase resulted with better dock score and energy for compound **1** as compared to standard drugs streptomycin, ciprofloxacin, ampicillin and fluconazole, that supported the wet lab results. Among the synthesized compounds, the compound **1** possessed good antioxidant activity as compared to standard L-ascorbic acid. The resultant data for antimicrobial activity of aesculin derivatives revealed compound **1** as the most potent antimicrobial compound as compared to the standard drugs streptomycin, ciprofloxacin, ampicillin and fluconazole. While compound **2** showed better antimicrobial activity as compared to streptomycin, ciprofloxacin, ampicillin. The preservative efficacy test for compound **1** in aloe vera juice and white lotion USP has been showed the log CFU/mL values within the prescribed limit of USP standard and results were comparable to standard sodium benzoate, ethyl paraben and propyl paraben. Compound **1** has been found to be within prescribed limit of stability study over six month.

**Conclusion:**

Compound **1** showed the potent G-6-P synthase inhibitory, antioxidant, antimicrobial, preservative efficacy and stability study results as compared to standard drugs taken. The results have found comparable to molecular docking results, and this final compound may be used as new preservatives for food and pharmaceutical products. Moreover, the mechanistic insight into the docking poses was also explored by binding interactions of aesculin derivatives inside the pdb id 1moq. These results also supported the results for novel synthesized G-6-P synthase inhibitors.

**Graphical abstract:**

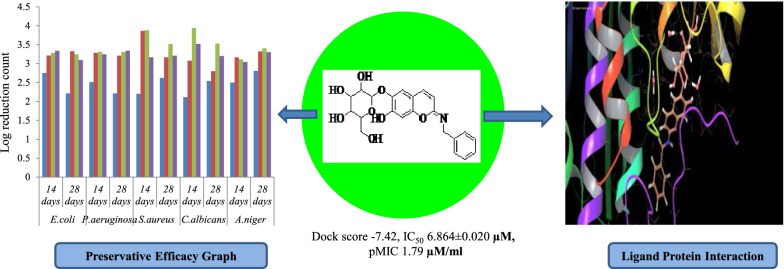

**Supplementary Information:**

The online version contains supplementary material available at 10.1186/s13065-021-00769-8.

## Introduction

**Fig. 1:**
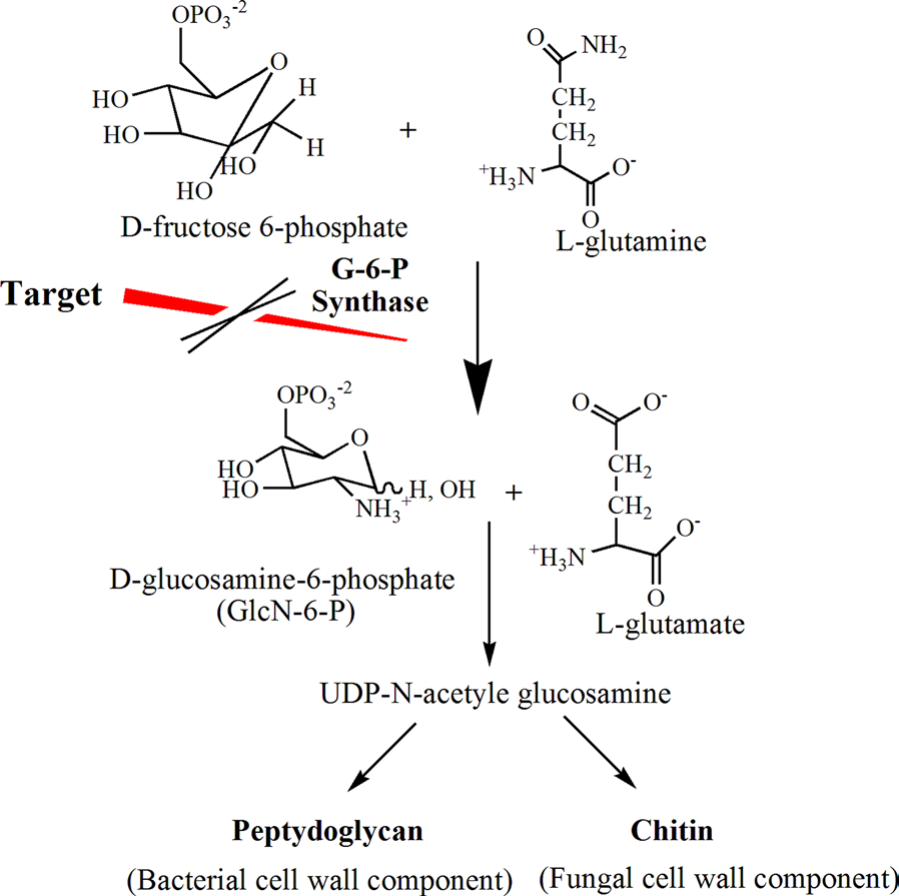
Role of G-6-P synthase in microbial cell wall synthesis

Glucosamine-6-Phosphate synthase (G-6-P synthase) catalyzed the first step in hexosamine biosynthesis and converted Fructose-6-Phosphate (Fru-6-P) into GlcN-6-P (Glucosamine-6-Phosphate), a precursor of Uridine Diphosphate N-acetyl glucosamine (UDP-NAG). NAG is an essential constituent of the peptidoglycan layer of the microbial cell wall as shown in Fig. 1. Accordingly, G-6-P synthase offers a potential target for the action of antimicrobial agents and hence, it has attracted the interest of several researchers [1].

Moreover, the reported literature revealed for the adverse effects of exiting antimicrobial based preservatives v*iz.* estrogenic effect, contact eczema, endocrine disruption, various types of cancer, etc. Hence, the search of better, and safe preservatives for food, pharmaceutical and cosmetic products seems necessary [2, 3].

The plants contains wide variety of phytoconstituents with diverse structural heterogeneity as compared to synthetic compounds and are also considered as an important source of novel and safe therapeutic agents [4]. The 3D-crystallographic structure of enzyme involved in microbial cell wall synthesis i.e. G-6-P synthase is available and can be explored as a novel target for search of better antimicrobial compounds [5]. Moreover, the phytoconstituents of plants with reported antimicrobial efficacy may be explored using the molecular docking and large number of molecules can be screened within a short time [6]. Further, the docking simulations if combined with synthetic medicinal chemistry to modify the phytoconstituents, it may help to design highly potent G-6-P synthase inhibitors. Several plant based G-6-P synthase inhibitors such as catechin, luteolin, etc. have been reported for their antimicrobial efficacy [7–9]. Hence, similar to this there is a scope to explore the other selected phytoconstituents for discovery of safe and better G-6-P synthase inhibitors.

Aesculin, β-d-glucose-6,7-dihydroxy coumarin is a compound derived from the horse chestnut tree has been associated with different biological properties such as anti-oxidant, photo-protective, inhibition of oxidative DNA damage, chemo-preventive, gastro protective, anti-tumor, etc. [10–12].

It has also been reported in our previous study that aesculin can act as an active inhibitor of G-6-P synthase enzyme based upon the results of molecular docking and ADMET data [13]. Hence, it was planned to explore various derivatives of aesculin for their antimicrobial, antioxidant, preservative efficacy and stability behavior to compare the wet lab results with that of the results of molecular docking.

## Experimental

### Materials and methods used

All the chemicals and reagents used in the experimental part of the study were of analytical grade. Nutrient agar, nutrient broth, sabouraud dextrose agar, and sabouraud broth were purchased from HiMedia Laboratories (Mumbai, India). Aesculin, 2,2-diphenyl-1-picrylhydrazyl (DPPH), anilines, hydrochloric acid were purchased from Sigma Aldrich (Germany), LobaChemie (Mumbai, India), and SRL (Mumbai, India). Reaction progress was checked by thin-layer chromatography (TLC) method. Standard streptomycin, ampicillin, ciprofloxacin, and fluconazole were obtained from Belco Pharma, Bahadurgarh (India). The standard microbial strains *E. coli* 45, *S. aureus* 3160, *P. aeruginosa* 1934, *C. albicans* 183 and *A. niger* 282 were obtained in lyophilized form from MTCC, Chandigarh (India). Melting point was recorded by the Sonar melting point apparatus. FTIR spectra were recorded on Perkin Elmer FTIR spectrophotometer, ^1^H NMR, and ^13^C NMR spectra were recorded on Bruker Avance II 400 NMR spectrometer. Mass spectra were recorded on Waters Micromass Q-ToF Micro instrument while the elemental analysis was done by Perkin Elmer 2400 elemental analyzer.

### Molecular docking

The three-dimensional structures of aesculin derivatives were constructed by using Chemdraw ultra 8, and energy was minimized with the LigPrep tool of Schrodinger Maestro. The X-ray crystal structures of G-6-P synthase were downloaded from the Protein Data Bank (http://www.rcsb.org/pdb). PDB ID 1MOQ (resolution of 1.57 Å) was selected on the basis of the lowest resolution as well availability and water molecules (except those coordinated to metals and between the ligand–protein) were removed with the help of Schrodinger protein preparation wizard [14]. The energy-restrained of the protein structure target site optimization of targeted protein G-6-P synthase was done by using Optimized Potential for Liquid Simulations (OPLS-2005) as force field. The partial charges were computed according to the OPLS-2005 force field (32 stereoisomers, tautomers, and ionization) on biological pH. All the calculations were carried out by using Schrodinger, Inc. (New York, USA) software Maestro 11 with an induced fit docking (IFD) method. The ligands prepared after energy minimization was used for molecular docking studies. All the computational work was performed in Laboratory for preservation technology and Enzyme Inhibition Studies, Department of Pharmaceutical Sciences, M.D. University, Rohtak, INDIA, was used for all computational work [15, 16].

### ADMET analysis

Quick prop from Schrodinger was utilized for *in-silico* prediction of ADME properties of proposed and synthesized aesculin derivatives. Various ADME parameters were calculated such as Log P, number of rotatable bonds, number of hydrogen acceptor, Log BB, and number of hydrogen bond donor atoms. Lipinski’s rule of five was also used for the prediction of a drug-like profile of newly synthesized derivatives.

### Procedure for synthesis of aesculin derivatives

**Fig. 2:**
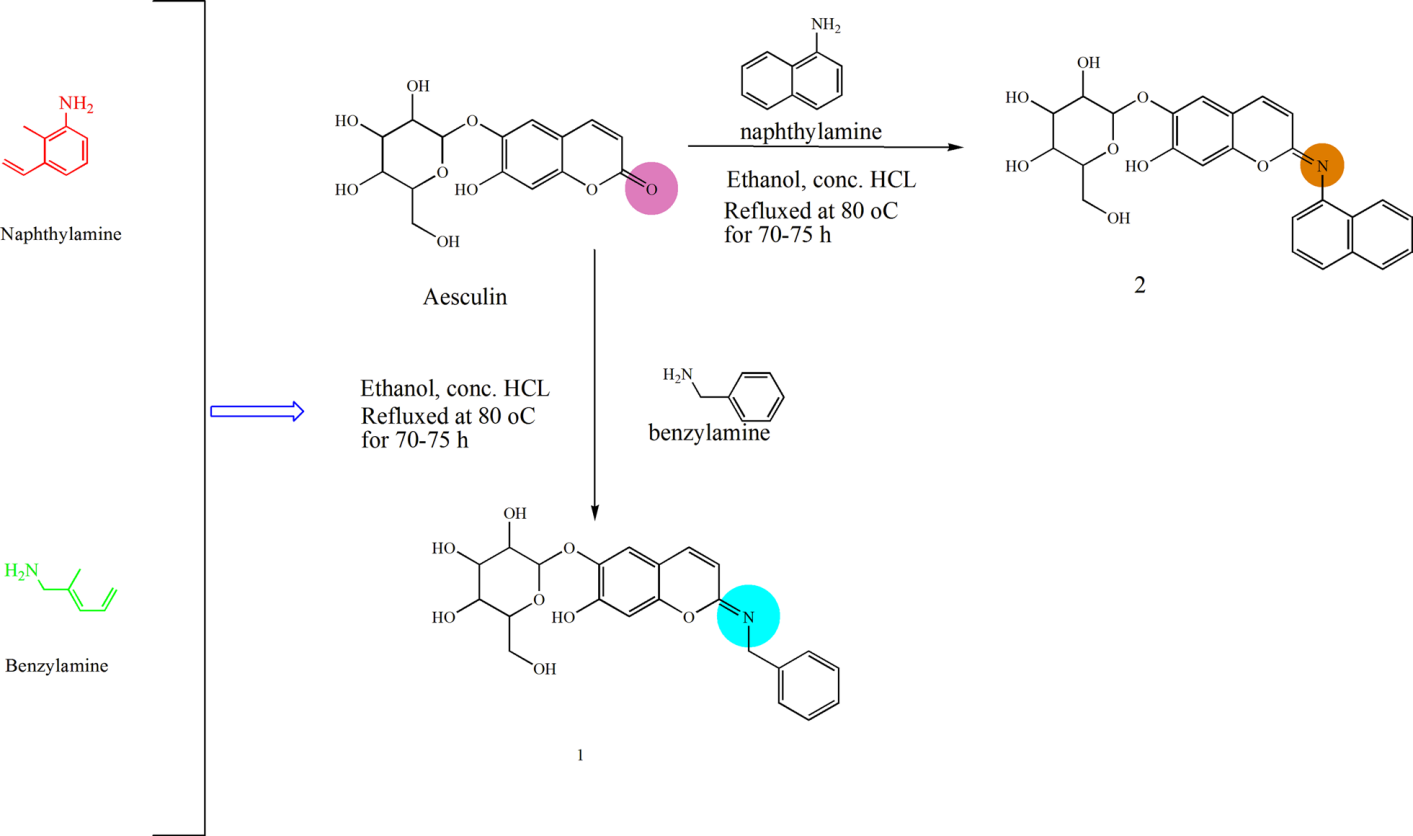
Design strategy and Scheme for the synthesis of aesculin derivatives

Aesculin derivatives were synthesized by some modifications in the procedure of Yang et al*.* 2006 as outlined in Fig. 2 [17]. The proposed derivatives were synthesized with substituted aniline (0.01 mol) taken in a round bottom flask. To this reaction mixture, concentrated hydrochloric acid was added dropwise with continuous stirring. Aesculin (0.01 mol) was dissolved in ethanol (50 mL) in equimolar concentration and was refluxed. Synthesis of derivatives was monitored by single spot TLC. On the completion of reaction the concentrated reaction mixture was precipitated. Recrystallization of the crude products was done by using alcohol. The final structure of the compounds was confirmed by FTIR, ^1^H NMR spectra, ^13^C NMR spectra, mass spectra and elemental analysis.

### Spectral data

#### 2-(3,4-dihydroxyphenyl)-3-(3-nitrophenylamino)chroman-5,7-diol (Compound 1)

*R*_*f*_ TLC mobile phase: Methanol: Chloroform (20:80) = 0.56; Yield = 35%; M.P. = 220–222 °C; M.Wt. = 429.14; IR (KBr pellets) cm^−1^: 1383 (–C–O–C), 1040 (–C–C–), 1684 (–C=N–), 2948 (–C–H–), 3387 (–OH–); ^1^H NMR (400 MHz, CDCL_3_): δ 9.93 (s, 1H), 7.66 (d, *J* = 9.2 Hz, 1H), 7.52 (d, *J* = 8.5 Hz, 1H), 7.44 (s, 1H), 7.35 (d, *J* = 6.0 Hz, 2H), 7.30 (d, *J* = 8.4 Hz, 2H), 7.28 (t, *J* = 8.0 Hz, 1H), 6.87 (s, 1H), 5.04 (s, 1H), 4.95 (d, *J* = 8.1 Hz, 1H), 4.62 (d, *J* = 7.2 Hz, 1H), 4.59 (d, *J* = 9.7 Hz, 1H), 4.52 (d, *J* = 10.6 Hz, 1H), 4.13 (d, *J* = 8.0 Hz, 1H), 4.07 (d, *J* = 7.8 Hz, 1H), 3.73 (dd, *J* = 13.8, 7.5 Hz, 1H), 3.50 (q, *J* = 9.3, 8.8 Hz, 1H), 3.41 (s, 2H), 3.38 (q, *J* = 8.5 Hz, 1H), 3.25–3.23 (m, 1H); ^13^C NMR (400 MHz, CDCL_3_) δ 160.24, 149.30, 147.84, 145.58, 145.08, 137.34, 129.17, 127.71, 126.97, 114.97, 112.87, 111.52, 102.93, 101.32, 79.51, 74.14, 73.41, 54.69, 38.44, 25.96, 12.61, 10.1; MS ES + (ToF): m/z 429.14 [M^+^+2]; CHNS: Calc (C_23_H_23_NO_8_): C, 61.53; H, 5.40; N, 3.26; O, 29.81; Found C, 61.54; H, 5.42; N, 3.27; O, 29.78.

#### 2-(3,4-dihydroxyphenyl)-3-(naphthalen-1-ylamino)chroman-5,7-diol (Compound 2)

*R*_*f*_ TLC mobile phase: Methanol: Chloroform (20:80) = 0.60; Yield = 40%; M.P.; 168–170 °C; M.Wt. = 465.14;; IR (KBr pellets) cm^−1^: 1166 (–C–O–C), 1077 (–C–C–), 1457 (–C = C–), 1699 (–C=N–), 2936 (–C–H–), 3390 (–OH–); ^1^H NMR (400 MHz, CDCL_3_) δ 9.93 (s, 1H), 8.32 (d, *J* = 7.3 Hz, 1H), 7.95 (d, *J* = 8.9 Hz, 2H), 7.86 (d, *J* = 8.4 Hz, 1H), 7.64 (d, *J* = 8.1 Hz, 1H), 7.56 (d, *J* = 10.8 Hz, 2H), 7.54 (d, *J* = 8.1 Hz, 1H), 7.31 (s, 1H), 7.13 (d, *J* = 8.6 Hz, 1H), 6.75 (s, 1H), 5.04 (s, 1H), 4.95 (d, *J* = 8.1 Hz, 1H), 4.62 (d, *J* = 7.6 Hz, 1H), 4.13 (d, *J* = 8.1 Hz, 1H), 4.07 (d, *J* = 8.6 Hz, 1H), 3.73 (dd, *J* = 13.8, 7.5 Hz, 1H), 3.50 (q, *J* = 8.9 Hz, 1H), 3.41 (s, 2H), 3.38 (q, *J* = 8.5 Hz, 1H), 3.25–3.23 (m, 1H); ^13^C NMR (400 MHz, CDCL_3_) δ 163.20, 149.30, 146.42, 146.35, 145.76, 145.58, 133.27, 129.24, 128.14, 128.06, 126.64, 126.56, 123.70, 122.78, 122.06, 112.87, 111.70, 110.94, 103.36, 101.62, 77.41, 75.43, 73.23, 70.74, 62.21; MS ES + (ToF): m/z 467.16 [M^+^+2]; CHNS: Calc (C_25_H_23_NO_8_): C, 64.51; H, 4.98; N, 3.01; O, 27.50; Found C, 64.53; H, 4.97; N, 3.02; O, 27.47.

### In vitro evaluation of antioxidant potential of synthesized derivatives of selected leads using DPPH method

The ability of the synthesized aesculin derivatives to scavenge DPPH radicals was determined by DPPH free radical scavenging method. The aliquot of test compounds at different concentrations in methanol was mixed. The different concentration used for the evaluation antioxidant potential includes 12.5, 25, 50, 75 and 100 μg/mL. The 0.1 mM solution of DPPH was prepared in methyl alcohol, and 1 mL of this solution was further diluted to 3 mL both for the sample and standard. After 30 min of incubation in darkness and at ambient temperature, the resultant absorbance was recorded at 517 nm. The tests were performed in triplicate and the % inhibition of compounds was calculated by using the formula:

$$\% {\text{ Inhibition }} = \, \left( {{\text{A}}_{{\text{c}}} {-}{\text{ A}}_{{\text{s}}} } \right) \, \times { 1}00/{\text{A}}_{{\text{c}}}$$

Here, A_c_ was the absorbance of the control, and A_s_ was the absorbance of the sample [18].

### In vitro evaluation of antimicrobial potential of synthesized derivatives of selected leads by using tube dilution method

The newly synthesized aesculin derivatives were further evaluated for their antimicrobial potential against various MTCC strains viz*. E. coli* 45, *P. aeruginosa* 1934, *S. aureus* 3160, *P. mirabilis* 3310*, A. niger* 282 and *C. albicans* by broth dilution method. The highest dilution of the test compound resulting in no growth of microorganism was recorded as their MIC value. Dilutions of test and standard compounds were prepared in double strength nutrient broth I.P. (bacteria) or Sabouraud dextrose broth I.P. (fungi) [19]. A 0.9% NaCl solution was used to adjust the turbidity of bacterial and fungal cultures. The CFU and density of microorganism was adjusted to 0.5 McFarland standards with the help of distilled water [20]. The samples were incubated at 37 °C for 24 h (bacteria), at 37 °C for 7 days (*A. niger*), and at 37 °C for 48 h (*C. albicans*), and the results were recorded in pMIC.

### Evaluation of preservative efficacy of selected antimicrobial/antioxidant derivatives

White lotion USP and Aloe vera juice was used for evaluation of preservative efficacy of the selected aesculin derivatives. Selected derivatives of aesculin were used as preservatives in equivalent amount in cosmetic and the food product [21]. Aloe vera juice was prepared as per the method described by Ahlawat et al. with slight modifications. The aloe vera juice thus obtained was used for the testing of food preservative efficacy [22, 23]. White lotion USP was prepared as per the method of Narang et al. The compounds **1** and **2** in equimolar amount (0.0013 mol of methyl paraben) were used as novel preservatives by replacing standard preservatives sodium benzoate, methyl paraben and propyl paraben in both the preparations [24]. Standard strains of *P. aeruginosa* 1934, *S. aureus* 3160, *E. coli* 45, *A. niger* 282, and *C. albicans* 183 from MTCC were used as common microbial contaminants for evaluation of a preservative efficacy as per the protocol described in I.P., 2010 [25].

### Test procedure

Aloe vera juice and White lotion USP were taken for preservative efficacy study and compound **1** was added as test preservative in equimolar quantity (0.0013 mol of methyl paraben) to that of standard preservative. A microbial cell count of 1 × 10^5^–1 × 10^6^ cfu/mL was used for microbial inoculation in a quantity of 0.5–1% to the volume of the product taken for study. Samples were incubated at room temperature for 28 days. On incubation the CFU/mL of the product was determined at 0 day, 7 days, 14 days, 21 days, and 28 days by using agar pour plate technique [26]. As per the USP standard protocol the log values of cfu/mL was calculated as not less than 2.0 log reduction from initial count at 14^th^ day of incubation and no increase in microbial count from 14th day to 28th days for fungi [27].

### Stability study of the selected preservatives as per ICH guidelines

From the results of preservative efficacy study, compound **1** was selected for further evaluation of its stability behavior as per the ICH guidelines. The compound 1 was added in the final containers containing the preparations of Aloe vera juice and White Lotion USP. Both the preparations having standard preservative and the test compound **1** were stored at 40° ± 2 °C at 75% RH ± 5% RH (as per ICH guidelines) and were analyzed for the change in pH and cfu/ml at the time interval of 0, 1, 2, 3, 4, 5 and 6 months.

### Statistical analysis

All the data was represented as mean ± standard deviation (SD) for three triplicates of each sample. One-way ANOVA test at a significance level of 0.05 (p < 0.05) using MS excel statistical tool was used to analyze the experimental data.

## Results and discussion

### Docking study

**Fig. 3:**
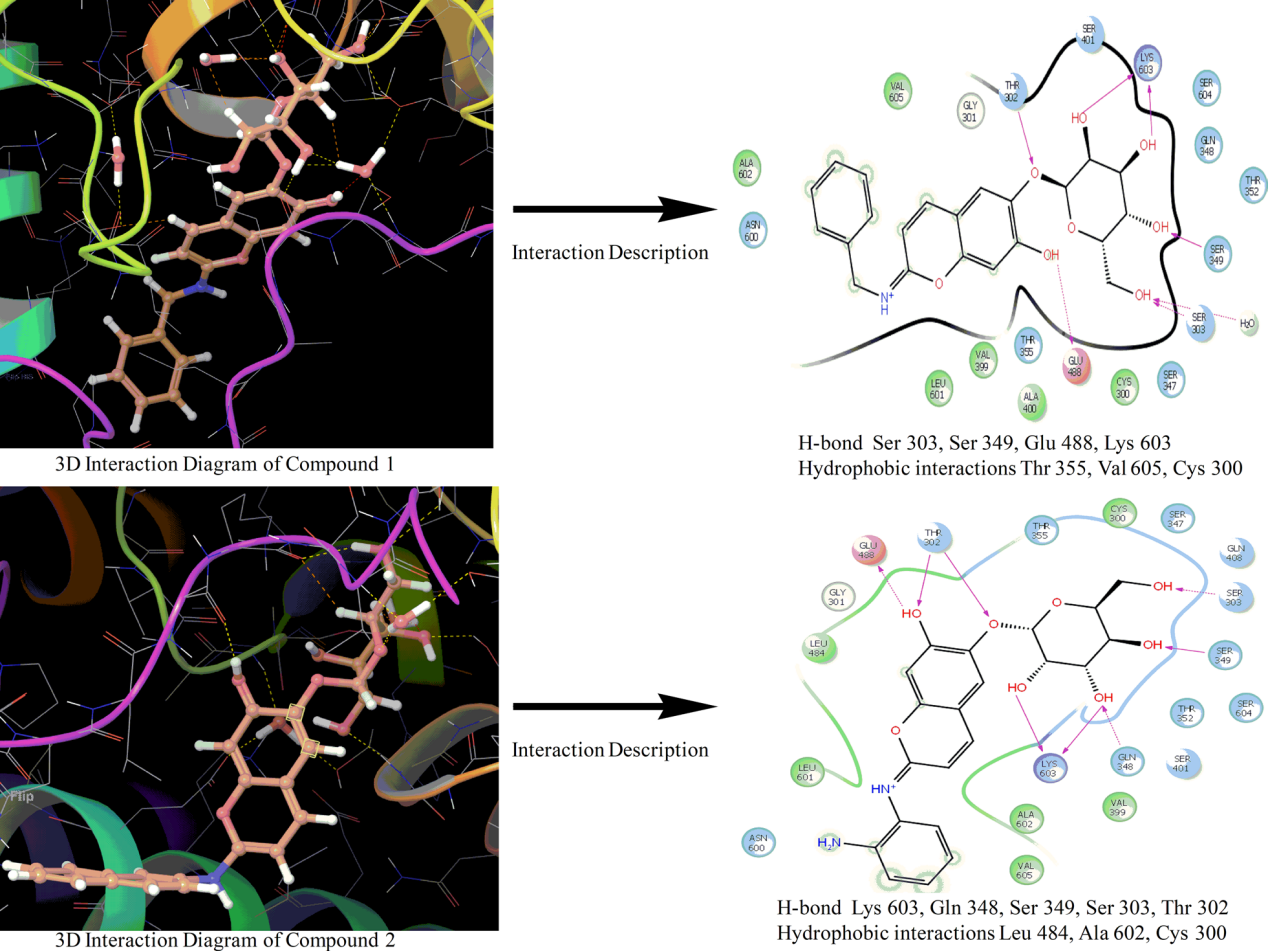
Binding pattern of compound **1** and** 2** with G-6-P synthase

The molecular docking of the proposed aesculin derivatives with the target site of pdb id 1moq showed that all the inhibitors were exhibiting better binding with different amino acids in active pocket of the enzyme G-6-P synthase. Binding pattern of compound **1** and** 2** with G-6-P synthase has been shown in Fig. 3.

The compound **1** and compound **2** were selected as the most active compounds based upon the dock score, binding energy, and ADMET parameters. Compound **1** exhibited better dock score (− 10.972) and binding energy (− 68.466 kJ/mol) values as compared to the dock scores (− 5.18, − 5.06, − 5.12) and binding energies (-37.16 kJ/mol, − 25.41 kJ/mol and − 23.15 kJ/mol) of standard drugs ciprofloxacin, ampicillin, and fluconazole respectively. Similar results were also reported for compound **2** as well and molecular docking results of different ligands within the binding pocket of enzyme G-6-P synthase environment have been shown in Table 1 [28].

**Table 1:** Docking score, binding energy showed by aesculin derivatives in comparison to standard antimicrobials

Compound(s)	Structure	G-6-P synthase binding affinity	
		Docking score	Energy (kJ/mol)
Compound 1		*− 10.972*	*− 68.466*
Compound 2		*− *8.144	*− *56.030
Aesculin		*− *7.037	*− *59.453
Standards	*Ciprofloxacin*	*− 5.185*	*− 37.163*
	*Ampicillin*	*− 5.065*	*− 25.411*
	*Fluconazole*	*− 5.129*	*− 23.156*

### ADMET analysis

The aesculin derivatives exhibited a suitable drug-like profile and the evaluation of different ADMET parameters have been represented in Table 2. Aesculin derivatives showed the value of various descriptors like rotatable bonds should be ≤ 10, QPlogBB should be > 1.0, and QPPCaco cell permeability should be in a range from 4 to 70 [29–31].

**Table 2:** Different ADMET parameters of aesculin derivatives

Compound(s)	ADMET profile						
	No. of rotatable bond	DonorHB	AcceptHB	QPlogPo/w	QPlogBB	QPPMDCK	QPPCaco
Compound 1	1	5	11	0.648	*− *2.227	36.288	89.19
Compound 2	2	6	12	*− *0.22	*− *2.842	9.9130	26.85
Aesculin	2	4	10	*− *0.21	*− *3–21	23.33	21.21
Streptomycin	9	12	15	*− *2.06	*− *4.20	0.78	3
Ciprofloxacin	3	2	6	*− *1.02	2.23	0.80	4
Ampicillin	4	3	5	*− *1.35	0.99	0.90	0.89
Fluconazole	5	1	5	*− *2.32	0.88	0.87	0.93

### Chemistry

Figure 2 with slight modifications was used for the synthesis of aesculin derivatives. With reference to FTIR data formation of compound **1** and **2** was confirmed by peak shifted and also confirmed by the ^13^C spectra. The change in chemical shift value, coupling constant and multiplicities were analyzed by ^1^HNMR protons signals of synthesized compounds. Mass spectra were also analyzed for the final confirmation of the synthesized compounds. Elemental analysis also confirmed the synthesis of aesculin derivatives where the percentages of C, H and N in synthesized compounds **1–2** was observed within defined limits. The FTIR, ^1^H NMR, ^13^C NMR, mass spectroscopy, and elemental analysis data confirmed the chemical structures of synthesized aesculin derivatives (Additional file 1).

### Antioxidant potential

The DPPH free radical scavenging assay confirmed that compound **1** possessed good antioxidant activity with inhibitory concentration IC_50_ 6.531 ± 0.042 µM as compared to standard L-ascorbic acid IC_50_ value, 8.110 ± 0.069 µM. The results of compounds **2** indicated a moderate antioxidant activity with IC_50_ 7.513 ± 0.076 µM while the aesculin itself has antioxidant potential with IC_50_ 7.513 ± 0.076 µM.

### Antimicrobial potential

The resultant data for antimicrobial activity of above mentioned aesculin derivatives revealed compound **1** as the most potent antimicrobial compound (pMIC values 1.53, 1.23, 1.23 and 2.13 µM/mL for *P. mirabilis, S. aureus* and *E. coli* respectively) as compared to the standard drugs streptomycin (pMIC values 1.06, 1.06 and 1.96 µM/mL for *P. mirabilis, S. aureus* and *E. coli* respectively), ciprofloxacin (pMIC values 1.12, 1.12 and 1.42 µM/mL for *P. mirabilis, S. aureus* and *E. coli* respectively), ampicillin (pMIC values 1.14, 0.84 and 1.74 µM/mL for *P. mirabilis, S. aureus* and *E. coli* respectively) and fluconazole (pMIC values 1.83 and 1.53 µM/mL for *C. albicans* and *A. niger* respectively). While compound **2** showed better antimicrobial activity (pMIC values 1.26, 1.26, 1.57 and 1.57 µM/mL for *P. mirabilis, P. aeruginosa, S. aureus* and *E. coli* respectively) as compared to streptomycin (pMIC values 1.06 and 1.06 µM/mL for *P. mirabilis* and *S. aureus* respectively), ciprofloxacin (pMIC values 1.42 and 1.42 µM/mL for *P. mirabilis* and *S. aureus* respectively), ampicillin (pMIC values 1.14, 0.84 and 0.84 µM/mL for *P. mirabilis, P. aeruginosa* and *S. aureus* respectively). Result of antimicrobial activity of aesculin derivatives (pMIC values) has been presented in Table 3**.** The result of antimicrobial activity revealed that the synthesized compounds have antimicrobial potential as compared to standards drugs. The probable mechanism of antimicrobial activity of aesculin derivatives may be due to the better inhibition of G-6-P synthase.

**Table 3:** Result of antimicrobial activity of aesculin derivatives (pMIC values)

Compound(s)	pMIC values in µM/mL					
	*P. mirabilis*	*P. aeruginosa*	*S. aureus*	*E. coli*	*C. albicans*	*A. niger*
Compound 1	*1.53*	*1.23*	*1.23*	*2.13*	*1.83*	*1.53*
Compound 2	1.26	1.26	1.26	1.57	1.57	0.96
Aesculin	< 0.83	< 0.83	< 0.83	< 0.83	< 0.83	< 0.83
Streptomycin	*1.06*	*1.36*	*1.06*	*1.96*	*–*	*–*
Ciprofloxacin	*1.12*	*1.42*	*1.12*	*1.42*	*–*	*–*
Ampicillin	*1.14*	*0.84*	*0.84*	*1.74*	*–*	*–*
Fluconazole	*–*	*–*	*–*	*–*	*1.08*	*1.38*

### Preservative efficacy

**Fig. 4:**
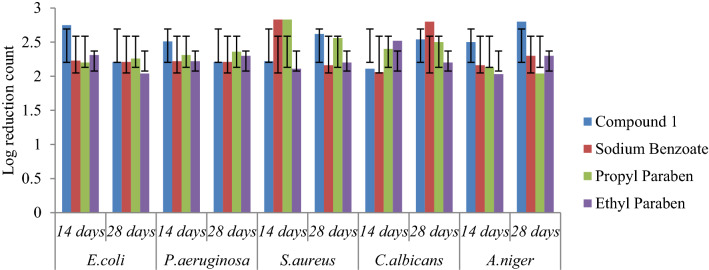
Preservative efficacy results of compound **1** in Aloe vera juice and degree of microbial log reduction

**Fig. 5:**
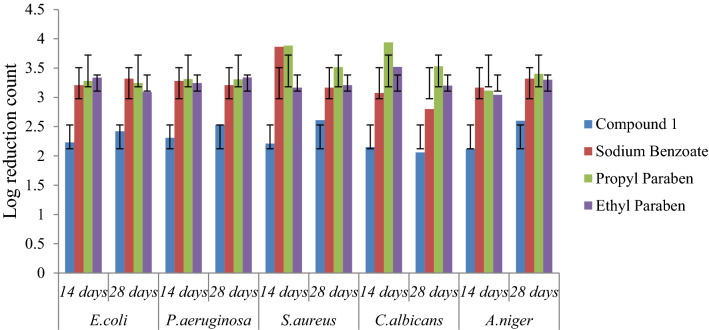
Preservative efficacy results of compound **1** in White Lotion USP and degree of microbial log reduction

Preservative efficacy study of the Aloe vera juice and White lotion USP were completed in triplicate and the results were reported as mean ± standard deviation. The result of log CFU/ml for compound **1** revealed that the values were within the prescribed limit as per USP criteria. The selected compound **1** reduced the growth of microbes on the 14th day from the initial count and found to be effective on the 28^th^ day, and results were also comparable to sodium benzoate (Figs. 4, 5). Preservative efficacy study results of compound **1** in Aloe vera juice and White lotion USP has been presented in Table 4.

**Table 4:** Log CFU/ml values of the selected compound **1** in Aloe vera juice and White lotion USP

Compound		*E. coli*		*P. aeruginosa*		*S. aureus*		*C. albicans*		*A. niger*	
Cfu/ml after days		14 days	28 days	14 days	28 days	14 days	28 days	14 days	28 days	14 days	28 days
Compound 1	#	2.75 ± 0.01^a^	2.21 ± 0.02^b^	2.51 ± 0.03^c^	2.21 ± 0.04^d^	2.22 ± 0.06^e^	2.62 ± 0.06^f^	2.11 ± 0.08^ g^	2.54 ± 0.02^ h^	2.50 ± 0.04^i^	2.80 ± 0.04^j^
	@	2.23 ± 0.01^a^	2.42 ± 0.03^b^	2.31 ± 0.04^c^	2.53 ± 0.03^d^	2.21 ± 0.04^e^	2.61 ± 0.03^f^	2.15 ± 0.06^ g^	2.06 ± 0.01^ h^	2.12 ± 0.02^i^	2.60 ± 0.02^j^
Sodium benzoate	*#*	*2.23* ± *0.01*^*a*^	*2.21* ± *0.01*^*b*^	*2.22* ± *0.01*^*c*^	*2.21* ± *0.02*^*d*^	*2.83* ± *0.01*^*e*^	*2.16* ± *0.01*^*f*^	*2.06* ± *0.02*^* g*^	*2.80* ± *0.02*^* h*^	*2.16* ± *0.02*^*i*^	*2.30* ± *0.01*^*j*^
	*@*	*3.23* ± *0.01*^*a*^	*3.33* ± *0.24*^*b*^	*3.22* ± *0.16*^*c*^	*3.21* ± *0.03*^*d*^	*3.83* ± *0.04*^*e*^	*3.16* ± *0.04*^*f*^	*3.07* ± *0.08*^* g*^	*2.80* ± *0.08*^* h*^	*3.16* ± *0.01*^*i*^	*3.32* ± *0.01*^*j*^
Propyl paraben	*#*	*2.20* ± *0.01*^*a*^	*2.26* ± *0.06*^*b*^	*2.31* ± *0.01*^*c*^	*2.36* ± *0.02*^*d*^	*2.83* ± *0.02*^*e*^	*2.56* ± *0.01*^*f*^	*2.40* ± *0.01*^* g*^	*2.50* ± *0.01*^* h*^	*2.13* ± *0.05*^*i*^	*2.04* ± *0.02*^*j*^
	*@*	*3.20* ± *0.57*^*a*^	*3.24* ± *0.36*^*b*^	*3.31* ± *0.01*^*c*^	*3.30* ± *0.01*^*d*^	*3.83* ± *0.02*^*e*^	*3.56* ± *0.01*^*f*^	*3.90* ± *0.02*^* g*^	*3.53* ± *0.01*^* h*^	*3.13* ± *0.06*^*i*^	*3.43* ± *0.01*^*j*^
Ethyl Paraben	*#*	*2.31* ± *0.03*^*a*^	*2.04* ± *0.18*^*b*^	*2.22* ± *0.16*^*c*^	*2.30* ± *0.11*^*d*^	*2.11* ± *0.04*^*e*^	*2.20* ± *0.01*^*f*^	*2.52* ± *0.02*^* g*^	*2.20* ± *0.02*^* h*^	*2.03* ± *0.02*^*i*^	*2.30* ± *0.05*^*j*^
	*@*	*3.36* ± *0.02*^*a*^	*3.00* ± *0.14*^*b*^	*3.24* ± *0.36*^*c*^	*3.34* ± *0.01*^*d*^	*3.16* ± *0.04*^*e*^	*3.10* ± *0.02*^*f*^	*3.50* ± *0.01*^* g*^	*3.20* ± *0.01*^* h*^	*3.03* ± *0.04*^*i*^	*3.30* ± *0.08*^*j*^

Initial microbial count present in inoculums 1 × 10^5^–1 × 10^6^; # Aloe vera juice; @ White lotion USP

CFU = Colony forming unit, all experiments were conducted in triplicate (n = 3) and the mean values are presented. Different letters mean p < 0.05 in each line by One-way ANOVA test

### Stability study

The results of six-months continuous stability testing were performed in triplicate and were reported as mean values. The pH of Aloe vera juice and White lotion USP samples were found to be in the range of 5.5–6.0, which indicated the stability of compound **1** as a preservative as compared to that of the standard preservatives sodium benzoate, propyl paraben, and methyl paraben. The results of the antimicrobial study also concluded the no microbial growth in samples containing compound **1** for 6 months period. These results of stability study showed that the products containing acesculin derivatives (compound 1) are stable for 6 months. Results of stability study have been shown in Table 5.

**Table 5:** Stability studies of compound **1** in aloe vera juice and White Lotion USP for pH

Compound(s)		Change in pH with time						
		0 month	1 month	2 month	3 month	4 month	5 month	6 month
Compound 1	#	5.8 ± 0.45^a^	6.0 ± 0.50^b^	5.9 ± 0.04^c^	5.7 ± 0.21^d^	5.8 ± 0.34^e^	5.9 ± 0.02^f^	5.8 ± 0.44^ g^
	@	5.6 ± 0.23^a^	5.9 ± 0.44^b^	5.7 ± 0.44^c^	5.8 ± 0.23^d^	5.7 ± 0.39^e^	5.8 ± 0.29^f^	6.0 ± 0.43^ g^
Sodium benzoate	**#**	5.6 ± 0.23	6.9 ± 0.44	5.7 ± 0.44	5.8 ± 0.23	6.7 ± 0.39	6.8 ± 0.29	7.0 ± 0.43
	**@**	5.7 ± 0.55	5.6 ± 0.89	5.6 ± 0.34	5.6 ± 0.46	6.0 ± 0.59	6.0 ± 0.93	5.6 ± 0.18
Propyl paraben	**#**	5.8 ± 0.07	5.7 ± 0.67	5.6 ± 0.67	5.7 ± 0.79	5.8 ± 0.35	5.9 ± 0.59	5.8 ± 0.28
	**@**	5.9 ± 0.56	5.6 ± 0.98	6.0 ± 0.89	5.8 ± 0.19	5.6 ± 0.18	5.5 ± 0.78	5.7 ± 0.82
Ethyl paraben	**#**	5.6 ± 0.23	6.9 ± 0.44	5.7 ± 0.44	5.8 ± 0.23	6.7 ± 0.39	6.8 ± 0.29	7.0 ± 0.43
	**@**	5.7 ± 0.55	5.6 ± 0.89	5.6 ± 0.34	5.6 ± 0.46	6.0 ± 0.59	6.0 ± 0.93	5.6 ± 0.18

^#^ Aloe vera juice; @ White lotion USP

All pH values were recorded in triplicate (n = 3) and the mean values are presented. Different letters mean p < 0.05 in each line by One-way ANOVA test

## Conclusion

From the results of antimicrobial study, preservative and stability study it can be concluded that the aesculin derivatives could act as G-6-P synthase inhibitors as the results also in correlation with molecular docking study. This correlation between different studies alo help in concluding the mechanism for the inhibition of G-6-P synthase with different visual binding interactions. The aesculin compound **1** showed superior DPPH scavenging potential, antimicrobial, better preservative efficacy results, and also able to prevent the pH changes and microbial CFU count in used food and pharmaceutical formulation. Therefore, the synthesized aesculin derivatives **1** can be used as novel and superior preservatives for food and pharmaceuticals.

## Supplementary Information

**Additional file 1.** Spectral Data File 1.

## Data Availability

The datasets used and/or analyzed during the current study are available from the corresponding author on reasonable request.

## Abbreviations

ADMET: Absorption, distribution, metabolism, excretion & toxicity
G-6-P Synthase: Glucosamine-6-phosphate synthase
CYP P450: Cytochromes P450
OATP1B1: Solute carrier organic anion transporter family member 1B1
DHEAS: Dehydroepiandrosterone
PPAR: Peroxisome proliferator-activated receptors
DPPH UDP-N-acetyl: 2,2-Diphenyl-1-picrylhydrazyl
Glucosamine: Uridine diphosphate *N*-acetylglucosamine
UDP-GlcNAc: UDP-*N*-acetyl glucosamine
Fru-6-P: d-Fructose-6-phosphate
GlcN-6-P: d-Glucosamine-6-phosphate
UDP-NAG: Uridine Diphospho-*N*-acetyl Glucosamine
FTIR: Fourier-transform infrared spectroscopy
IC50: Inhibitory concentration
MIC: Minimum inhibitory concentrations
CFU: Colony forming unit
HBA: Hydrogen bond acceptor
HBD: Hydrogen bond donor
MW: Molecular weight
MTCC: Microbial Type Culture Collection
DMSO: Dimethyl sulfoxide
BOD: Biological oxygen demand
PDB ID: Protein Data Bank Identification
OPLS: Optimized potential for liquid simulations
QS: Quantity sufficient
