# Supplementary material for: Aesculin based glucosamine-6-phosphate synthase inhibitors as novel preservatives for food and pharmaceutical products: in-silico studies, antioxidant, antimicrobial and preservative efficacy evaluation
Source: BMC Chem. 2021 Jul 27;15(1):45. doi: 10.1186/s13065-021-00769-8 (PMC8317424; doi:10.1186/s13065-021-00769-8)
Supplement: Supplementary file 1 — Additional file 1. Spectral Data File 1. [file 13065_2021_769_MOESM1_ESM.docx]

**Compound 1 – FTIR Spectra**

**Compound 1 –^1^H-NMR Spectra**

**
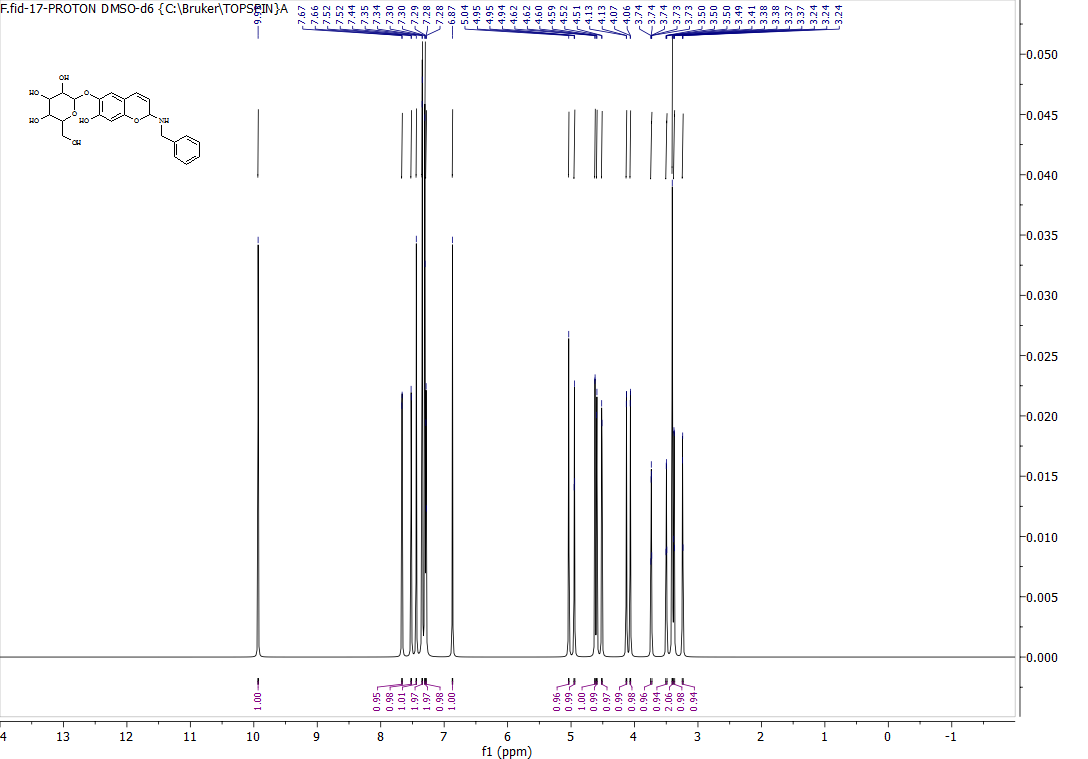
**

**Compound 1 –^13^C-NMR Spectra**

**Compound 2 – FTIR Spectra**

**Compound 2 –^1^H-NMR Spectra**

**
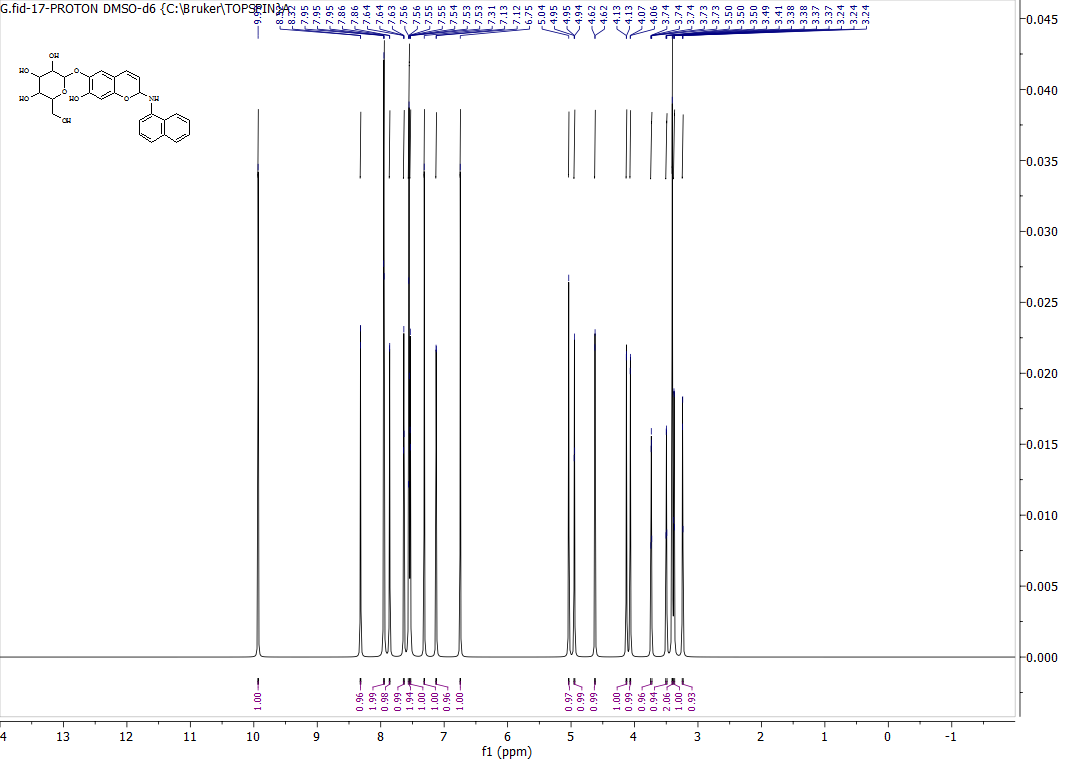
**

**Compound 2 –^13^C-NMR Spectra**
